# Bayesian Deconvolution for Angular Super-Resolution in Forward-Looking Scanning Radar

**DOI:** 10.3390/s150306924

**Published:** 2015-03-23

**Authors:** Yuebo Zha, Yulin Huang, Zhichao Sun, Yue Wang, Jianyu Yang

**Affiliations:** School of Electronic Engineering, University of Electronic Science and Technology of China, 2006 Xiyuan Road, Gaoxin Western District, Chengdu 611731, China; E-Mails: yulinhuang@uestc.edu.cn (Y.H.); threadkite@sina.com (Z.S.); wyue_uestc@126.com (Y.W.); jyyang@uestc.edu.cn (J.Y.)

**Keywords:** deconvolution, Bayesian, radar imaging, super-resolution, convex optimization

## Abstract

Scanning radar is of notable importance for ground surveillance, terrain mapping and disaster rescue. However, the angular resolution of a scanning radar image is poor compared to the achievable range resolution. This paper presents a deconvolution algorithm for angular super-resolution in scanning radar based on Bayesian theory, which states that the angular super-resolution can be realized by solving the corresponding deconvolution problem with the maximum a *posteriori* (MAP) criterion. The algorithm considers that the noise is composed of two mutually independent parts, *i.e.*, a Gaussian signal-independent component and a Poisson signal-dependent component. In addition, the Laplace distribution is used to represent the prior information about the targets under the assumption that the radar image of interest can be represented by the dominant scatters in the scene. Experimental results demonstrate that the proposed deconvolution algorithm has higher precision for angular super-resolution compared with the conventional algorithms, such as the Tikhonov regularization algorithm, the Wiener filter and the Richardson–Lucy algorithm.

## 1. Introduction

Due to the superiorities of microwave remote sensing over optical remote sensing, such as all-weather and all-timing imaging and long operating distance, microwave remote sensing has been widely used in many civilian and military fields [1–5]. The most popular microwave remote sensing system is radar, which achieves two-dimensional high resolution imaging via signal processing. The high range resolution can be obtained by transmitting wide-bandwidth waveforms and using the maximum autocorrelation or global optimization method [6]. As for the azimuth direction, the angular resolution can be obtained by Doppler beam sharpening or the synthetic aperture radar technique. However, these techniques are not suitable for forward-looking imaging in airborne radar and fixed ground-based radar, because the direction of the Doppler resolution and the range resolution are the same in the forward-looking area of the radar, which leads to the failure to use the frequency information [7]. One option to achieve high azimuth resolution in the forward-looking area of the radar platform is to separate the transmitting station and receiving station. This forms the bistatic SAR, which has many advantages in comparison with its monostatic counterpart [8]. However, there are several problems associated with bistatic SAR, including interferences from the geometry configuration and the complexity of the hardware.

Scanning radar imaging belongs to real aperture imaging, working as a noncoherent sensor. The high range resolution of scanning radar can be obtained using pulse compression. The upper limit of the angular resolution is determined by the effective wavelength and the size of the antenna, which is restricted by the radar system. Improvement in the angular resolution of the scanning radar image can be accomplished by increasing the physical size of the antenna. Increasing the size of the radar antenna is costly, because the forward-looking scanning radar is often mounted on an airborne platform, where there may be insufficient space to accommodate a large-sized antenna [9]. Hence, the angular resolution of the forward-looking scanning radar is usually poor when compared with the achievable range resolution.

Under the Born hypothesis [10], the echo in the azimuth can be modeled as the convolution of the transmitted signal with the reflectivity of the observed scene. Therefore, the deconvolution method can enhance the angular resolution of the scanning radar image in theory [11,12]. The data recorded at the output of the radar system are a low-pass-filtered version of the original scene due to the finite size of the antenna. The portions lost by the antenna are the high frequency spectral components [13]. Convolution in the Fourier domains corresponds to multiplication, while deconvolution is Fourier division [14]. The challenge is that the multipliers are often small for high frequencies, and division is unstable due to noise presented in the reflectivity data. This phenomenon also occurs in communication systems.

Deconvolution aims at removing the system noise and other non-idealities of the image processing and is inherently an ill-posed problem [15]. Basically, there are two main categories of deconvolution techniques: set-theoretic estimation methods [16–18] and Bayesian approaches. One of the popular attempts at employing a set-theoretic framework for deconvolution problem is the regularization method. Recently, the application of regularization deconvolution can be found in [19–23] for microwave imaging. One of the popular regularization methods is Tikhonov regularization [24]. The main motivation behind the regularization methods lies in replacing the original deconvolution problem with a nearby well-conditioned problem. A scheme that uses the singular value decomposition (SVD) has been reported in [25]. However, this method in the space domain is sensitive to the noise and loses performance in terms of super-resolution. Recently, the authors of [3] presented a truncated singular value decomposition (TSVD) algorithm to enhance the spatial resolution of radiometer data. The main idea of TSVD is to choose a truncation parameter so that all of the noise-dominated SVD coefficients are discarded, which makes application of TSVD for angular super-resolution in scanning radar challenging.

Another direction for the deconvolution problem that is receiving greater popularity is the use of Bayesian theory. Following the pioneering work [26,27], the Bayesian deconvolution algorithms have been studied extensively in radar imaging. In [28], the authors applied the Bayesian deconvolution algorithm for ISARimaging, resulting in better resolution, even under strong noise. It is also studied for ISAR imaging with sparse aperture in [29]. A Bayesian inversion approach to estimate Titan's lake features has also been presented in [30]. An important advantage of Bayesian deconvolution algorithms over the regularization deconvolution algorithms is, as proposedlater, Bayesian deconvolution algorithms can use the statistic characteristic of prior information about the solution, which is often ignored by the regularization methods. On the other hand, the Bayesian methods are model-based and can handle observation noise and missing data [31].

This paper presents a deconvolution algorithm for angular super-resolution in scanning radar using the Bayesian theory. To use the Bayesian theory for solving the deconvolution problem, we first convert the deconvolution problem into an equivalent MAP estimation task that not only incorporates the prior information of the targets in the spirit of [28,29], but also considers the mixture noise in the echo data. In the proposed algorithm, we assume that the noise is composed of two mutually independent parts, a Gaussian signal-independent component and a Poisson signal-dependent component [32–34]. The application of the Poisson distribution can be found in [35] for optical imagery, in [36] for position emission tomography and in [37] for single positron emission computer tomography. However, no work about the Poisson distribution has been studied for angular super-resolution in scanning radar imaging. The Poisson component models the signal-dependent part of the errors, which is essentially due to the working mode of the scanning radar, while the Gaussian component represents the signal-independent parts of the errors, such as the thermal and electric noise. This forms the key feature of our approach to model noise in the proposed algorithm. On the other hand, the most crucial point of applying Bayesian methods is the choice of the form of the prior distribution. Inspired by Bayesian compressed sensing [38], the Laplace distribution is applied to represent the statistical characteristics of prior information about the targets. This leads to the objective function of the MAP estimation problem consisting of the so-called data term, which is nonlinear, plus a convex non-differentiable regularizer (the negative log-prior). We solve the MAP estimation problem in the framework of convex optimization by using an approximation method. Experimental results with synthetic data indicate that the proposed deconvolution algorithm for angular super-resolution has higher precision compared with conventional deconvolution algorithms (Richardson–Lucy deconvolution algorithm, Tikhonov regularization algorithm and Wiener filter).

The rest of the paper is organized as follows. Section 2 presents the signal model of scanning radar and mathematically formulates the angular super-resolution problem as an equivalent deconvolution problem. Section 3 presents the Bayesian inversion approach for solving the deconvolution problem, including the likelihood function and prior information about the targets. In Section 4, experimental results with synthetic data indicate that the proposed deconvolution algorithm for angular super-resolution in scanning radar has higher precision compared to the conventional deconvolution algorithms. Finally, conclusions are drawn in Section 5.

## 2. Angular Super-Resolution Model

In this section, we mainly describe the angular super-resolution model with emphasis on the echo formulation and then derive the mathematical formulation of angular super-resolution in forward-looking scanning radar. The construction of forward model consists of two steps: (1) formulating the signal model of forward-looking scanning radar; and (2) converting the problem of angular super-resolution into an equivalent deconvolution task.

**Figure 1.**
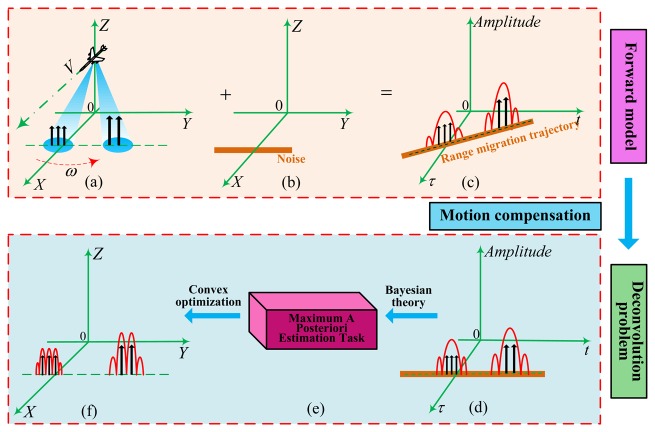
Diagram of forward-looking scanning radar used for angular super-resolution: (a) geometry model for scanning radar imaging; (b) noise; (c) data acquisition for scanning radar; (d) echo data after range com- pression and range cell migration; (e) deconvolution method; (f) angular super-resolution result.

Suppose that scanning radar works in forward-looking mode. The forward model of forward-looking scanning radar is illustrated at the top of Figure 1. The radar platform is moving along axis *X* corresponding to the range direction at altitude *H* with constant velocity *V*; the antenna beam scans the scene along the axis *X* corresponding to the azimuth direction with constant angular velocity ω and then receives the echo data from the observed scene. Firstly, conventional range compression and range cell migration are applied to the echo data with the current approaches [39].

Under the Born hypothesis [19,40], the data after range compression and range cell migration *g_R_* (*τ*, *t*) can be modeled as the convolution of the antenna beam *h* (*τ*, *t*) with the reflectivity coefficients of the observed scene *f* (*t*), which is:

(1) $$g_{R}\left(\tau ,t\right)=\int_{-\infty }^{+\infty }h\left(\tau ,t-t^{\prime }\right)f(t^{\prime })dt^{\prime }+n\left(\tau ,t\right)$$

where *τ* is the fast time, *t* is the slow time and *n* is the noise.

In the case of the observation of a certain scene *F*, the reflectivity coefficients of the scatters in the observed scene can be represented as a 2D matrix:

$$F=\left[\begin{array}{llll} f\left(x_{1},y_{1}\right), & f\left(x_{1},y_{2}\right) & \cdots & f\left(x_{1},y_{𝔄}\right)\\ f\left(x_{2},y_{1}\right), & f\left(x_{2},y_{2}\right) & \cdots & f\left(x_{2},y_{𝔄}\right)\\ \vdots & \vdots & \ddots & \vdots \\ f\left(x_{ℜ},y_{1}\right), & f\left(x_{ℜ},y_{2}\right) & \cdots & f\left(x_{ℜ},y_{𝔄}\right) \end{array}\right]$$

where *f* (*x_p_,y_q_*), (*p* = 1,2,⋯, *ℜ,q* = 1, 2,⋯, 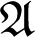) are the discrete equivalent backscattering coefficients at the p-th position of the range along the axis *X* at the *q-th* position along the azimuth of the observation scene, *ℜ* is the number of discretization cells of the observed scene along the *X* axis and 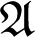 is the number of discretization cells of the observed scene along the *Y* axis. Each row of matrix *F* represents the backscatter coefficients index set in each range cell.

To denote the reflectivity coefficient of the observed scene, the 2*D* reflectivity coefficient matrix ***F*** should be reshaped to a column vector by stacking the rows of *F:*

(2) $$f={\left[f\left(x_{1},y_{1}\right),f\left(x_{1},y_{2}\right)\cdots f\left(x_{1},y_{𝔄}\right),f\left(x_{2},y_{1}\right)\cdots f\left(x_{2},y_{𝔄}\right),\cdots ,f\left(x_{ℜ},y_{1}\right)\cdots f\left(x_{ℜ},y_{𝔄}\right)\right]}^{T}$$

where f is a *ℜ* 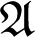 × 1 target vector and *ℜ* 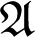 is the total number of targets after the discretization of the scene.

The 2D discrete time radar signal can be denoted as:

(3) $$\begin{array}{c} g_{e}\left(\tau_{r},t_{a}\right)=\overset{ℜ𝔄}{\underset{i=1}{\text{∑}}}f(i)\times \text{rect}\left(\frac{\tau_{r}-\frac{2R\left(t_{a},x_{p},y_{q}\right)}{c}}{T_{r}}\right)\\ r=1,2,\cdots ℜ;a=1,2,\cdots 𝔄 \end{array}\times \exp\left(-j\frac{4\pi f_{c}}{c}R\left(t_{a},x_{p},y_{q}\right)\right)$$

where *τ_r_* represents the *r*-th sample in fast time, *t_a_* is the *a*-th sample in slow time, *f*(*i*) is the *i*-th element in Equation (2), *rect*(·) is a rectangular function, *T_r_* denotes the pulse duration, *f_c_* is the carrier frequency, *c* is the speed of light, *ℜ* is the number of range samples and 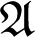 is the number of azimuth samples.

**Figure 2.**
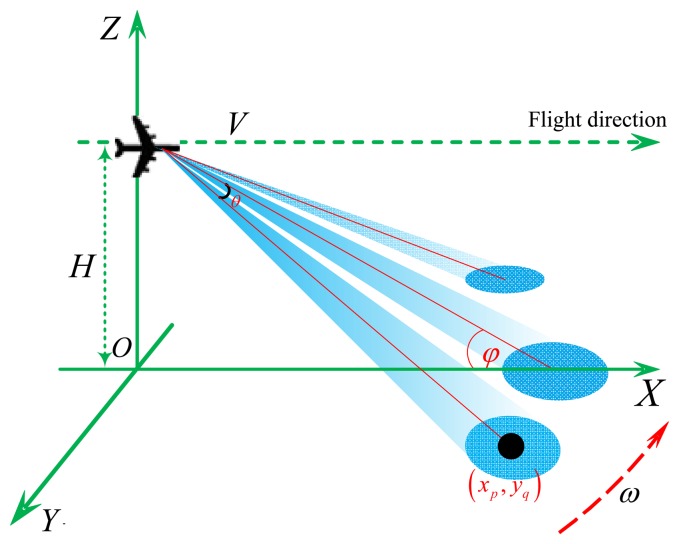
The geometry relationship of scanning radar.

The *R*(*t_a_,x_p_,y_q_*) is the range history between the antenna and a point target at (*x_p_,y_q_*) when the azimuth time is *t_a_*. The equation for *R (t_a_, x_p_, y_q_)* is shown in Equation (4)

(4) $$R\left(t_{a},x_{p},y_{q}\right)=\sqrt{R_{0}^{2}+{\left(Vt_{a}\right)}^{2}-2R_{0}Vt_{a}\cos\theta \cos\varphi }$$

in which *R*_0_ is the range when the antenna beam center is across the target at *(x_p_, y_q_), V* is the platform velocity, θ denotes the angle between the direction of the antenna and flight direction and φ represents the incident angle of the beam, which is usually smaller than 10°. The geometry relationship is illustrated in Figure 2.

At the time when the antenna beam center crosses the target at *(x_p_, y_q_)*, cubic and higher order terms of the Taylor expansion for Equation (4) can be ignored in the azimuth phase history of the targets [39]. In this case, Equation (4) can be simplified to:

(5) $$R\left(t_{a},x_{p},y_{q}\right)=R_{0}-\left(V\cos\theta \cos\varphi \right)t_{a}+\frac{V^{2}\sin^{2}\theta \sin^{2}\varphi }{2R_{0}}t_{a}^{2}+o(t_{a})$$

Since the product of the velocity *V* and azimuth time *t_a_* is much smaller than *R*_0_, Equation (5) can be rewritten as:

(6) $$R\left(t_{a},x_{p},y_{q}\right)\approx R_{0}-\left(V\cos\theta \cos\varphi \right)t_{a}$$

which leads to the range migration trajectory presented as a straight line in Figure 1c.

Substituting Equation (6) into Equation (3) and applying the range compression to the data *g_e_(τ_r_,t_a_)* yields:

(7) $$g_{c}\left(\tau_{r},t_{a}\right)=\sum_{i=1}^{ℜ𝔄}f(i)\times \delta \left(\tau_{r}-\frac{2R\left(t_{a},x_{p},y_{q}\right)}{c}\right)\times A\left(t_{a}\right)\times \exp\left(-j\frac{4\pi f_{c}}{c}R\left(t_{a},x_{p},y_{q}\right)\right)$$

where δ(·) is the impulse function and *A(t_a_)* is the antenna pattern.

After range cell migration correction to the data *g_c_* (*τ_r_,t_a_*), we have:

(8) $$g\left(\tau_{r},t_{a}\right)=\sum_{i=1}^{ℜ𝔄}f(i)H\left(\tau_{r},t_{a},x_{p},y_{q}\right)+n\left(\tau_{r},t_{a}\right)$$

where:

(9) $$H\left(\tau_{r},t_{a},x_{p},y_{q}\right)=\delta \left[B\left(\tau_{r}-\frac{2R_{0}}{c}\right)\right]\times A(t_{a})\times \exp\left\{-j\frac{4\pi f_{c}}{c}R\left(t_{a},x_{p},y_{q}\right)\right\}$$

and *B* is the signal bandwidth; the corresponding data *g(τ_r_,t_a_)* are shown in Figure 1d, and *n(τ_r_,t_a_)* denotes noise.

In Figure 1d, the amplitude of the received signal at the receiving output is proportional to the antenna pattern. If the two targets are close enough, the response of the two targets are proportional to two replicas of the antenna pattern, overlapped and added to get a composite response. Clearly, the space limitation at which targets are resolved is determined by the beam width of the antenna pattern. The resulting low-resolution signal is shown in Figure 1d. This phenomenon brings great difficulty in realizing the angular super-resolution imaging.

When we take the noise into account, Equation (8) can be written in matrix form as:

(10) g=H⊗f+n

where g and n are of dimension *ℜ* 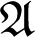 × 1, **H** is a *ℜ* 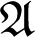 × *ℜ* 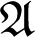 matrix and ⊗ indicates the convolution operator. In Equation (10):

(11) $$g={\left[g\left(\tau_{1},t_{t}\right),\cdots ,g\left(\tau_{1},t_{𝔄}\right),\cdots g\left(\tau_{𝔄},t_{t}\right),\cdots g\left(\tau_{ℜ},t_{𝔄}\right)\right]}^{T}$$

It can be shown in [1] that the convolution relationship holds between the expected value of |*g(τ_r_,t_a_)*|^2^ and |*f(x_p_,y_q_)*|^2^ with the two-way antenna power pattern |*a(t_a_)*|^2^. That is:

(12) $$E\left\{{\left|g\left(\tau_{r},t_{a}\right)\right|}^{2}\right\}={\left|A\left(t_{a}\right)\right|}^{2}⊗E\left\{{\left|f\left(x_{p},y_{q}\right)\right|}^{2}\right\}$$

where *e*{·} denotes the expected value operator. Therefore, the matrix **H** in Equation (10) is written as follows:

(13) $$H=\left[\begin{array}{lllll} H_{1} & H_{ℜ} & H_{ℜ-1} & \cdots & H_{2}\\ H_{2} & H_{1} & H_{ℜ} & \cdots & H_{3}\\ H_{3} & H_{2} & H_{1} & \cdots & \vdots \\ \vdots & & & & \\ H_{ℜ} & H_{ℜ-1} & H_{R-2} & \cdots & H_{1} \end{array}\right]$$

Each element of matrix **H** is the module operation result of *h* (*τ_r_,t_a_,x_p_, y_q_*). Therefore, the matrix **H** can be written as:

(14) $$H={\left[\begin{array}{ccccccc} H\left(\tau_{1},t_{1},x_{1},y_{1}\right) & \cdots & H\left(\tau_{1},t_{1},x_{1},y_{𝔄}\right) & \cdots & H\left(\tau_{2},t_{1},x_{1},y_{1}\right) & \cdots & H\left(\tau_{2},t_{1},x_{1},y_{𝔄}\right)\\ \vdots & & \vdots & \cdots & \vdots & & \vdots \\ H\left(\tau_{1},t_{𝔄},x_{1},y_{1}\right) & \cdots & H\left(\tau_{1},t_{𝔄},x_{1},y_{𝔄}\right) & \cdots & H\left(\tau_{2},t_{𝔄},x_{1},y_{1}\right) & \cdots & H\left(\tau_{2},t_{𝔄},x_{1},y_{𝔄}\right)\\ \vdots & \ddots & \vdots & \vdots & \vdots & \ddots & \vdots \\ H\left(\tau_{ℜ},t_{1},x_{ℜ},y_{1}\right) & \cdots & H\left(\tau_{ℜ},t_{1},x_{ℜ},y_{𝔄}\right) & \cdots & H\left(\tau_{1},t_{1},x_{1},y_{1}\right) & \cdots & H\left(\tau_{1},t_{1},x_{1},y_{𝔄}\right)\\ \vdots & & \vdots & \cdots & \vdots & & \vdots \\ H\left(\tau_{ℜ},t_{𝔄},x_{ℜ},y_{1}\right) & \cdots & H\left(\tau_{ℜ},t_{𝔄},x_{ℜ},y_{𝔄}\right) & \cdots & H\left(\tau_{1},t_{𝔄},x_{1},y_{1}\right) & \cdots & H\left(\tau_{1},t_{𝔄},x_{1},y_{𝔄}\right) \end{array}\right]}_{ℜ𝔄\times ℜ𝔄}$$

At this point, the goal of angular super-resolution imaging in forward-looking scanning radar is to infer, as accurately as possible, f from the samples g. This task is called the deconvolution problem in this paper.

A naive method would be simple division of data g by transferring the function in the Fourier domain. Using the Fourier transform, Equation (10) can be written as:

(15) G(w)=H(w)F(w)+N(w)

where **G**(*w*),**H**(*w*),**F**(*w*) and ***N***(*w*) are the Fourier transforms of g, **H**, **f** and **n**, respectively. The conventional deconvolution methods are that to find a linear operator **T**(*w*), such that:

(16) $$\hat{F}\left(w\right)=\frac{G\left(w\right)}{T\left(w\right)}=F\left(w\right)+\frac{N\left(w\right)}{H\left(w\right)}$$

where—denotes the matrix division operator. The challenge is that convolution in the Fourier corresponds to multiplication, and deconvolution is Fourier division. For the radar system, the multipliers are often small for high frequencies, and the inverse filter $\frac{1}{T\left(w\right)}$ is large for **T** (w) very small. This results in a large noise amplification and, thus, a poor angular resolution.

To address this challenge, we propose a Bayesian deconvolution method for angular super-resolution imaging in scanning radar in the next section.

## 3. Bayesian Inversion Approach for Angular Super-Resolution

The data g recorded at the output of the radar system are a low-pass-filtered version of the original data **f**. Thus, to recover the original scene from the recorded data g by the deconvolution approach, we firstly need to compensate for the loss of high-frequency information beyond the passband range. For this purpose, the obtained angular super-resolution model Equation (10) can be recast in the Bayesian framework. In this framework, we can handle prior information about the original scene and the likelihood function between the observation and the original scene.

Starting from Equation (10), we first transform the deconvolution problem into an equivalent MAP estimation task, and the corresponding problem can be equivalently transformed into an unconstrained optimization problem by adopting the negative logarithm of the posterior probability *p* (**f**|**g**). By Equation (10), the MAP estimation task consists of finding a solution that satisfies the following criterion:

(17) $$\begin{array}{ll} f_{\text{MAP}} & =\underset{\text{f}}{\text{arg max}p}\left(f|g\right)=\underset{\text{f}}{\text{arg max}p}\left(g|f\right)p(f)\\ & =\underset{\text{f}}{\text{arg max}}\left\{-\ln p\left(g|f\right)-\ln p(f)\right\} \end{array}$$

where *p* (**g**|**f**) represents the likelihood function pertaining to the angular super-resolution model Equation (10), which models all of the information coming from the data and their uncertainty, and *p* (**f**) is the prior probability density function (pdf) that models the information coming from the other source [41]. The prior term *p*(**f**) in Equation (17) is a function of f, which does not vary with the observation model.

In Equation (17), the − ln *p*(**g**|**f**) measures the violation of the relation between f and its observation **g**, and − ln p(**f**) corresponds to the prior information about the f, which does not vary with the measurement in the framework of Bayesian theory. It is noted that Equation (17) looks similar to the regularization method, because they are philosophically similar. The main idea behind these methods is to find a solution to the deconvolution problem [42]. However, the implementation of Equation (17) requires the knowledge of a likelihood pdf *p*(**g**|**f**) and the prior pdf p (f). The details on choosing these functions are shown as follows.

### 3.1. Likelihood

The choosing of the likelihood function mainly depends on the application. In radar signal processing [19,43], the likelihood function between the observation data and the original scene is approximated as a zero mean Gaussian distribution. However, there are problems with this assumption in angular super-resolution. The first is that such a continuous time process cannot exist, since it would have infinite power. For details about this reason, we refer the interested reader to [44]. In addition, the treatment of the likelihood function by the Gaussian distribution involves interesting ideas in the field of mathematical statistics [44]. In this paper, we assume that the noise is composed of two mutually independent parts, a Poisson signal-dependent component n*_p_* and a Gaussian signal-independent component n*_g_*. The Poisson component n*_p_* models the signal-dependent part of the errors, which is essentially due to the working mode of the scanning radar, while the Gaussian component n*_g_* represents the signal-independent parts of the errors, such as the thermal and electric noise.

In applications of angular super-resolution, where a high number of data are collected, the echo data are inherently affected by signal-dependent noise. Notice that the Poisson distribution is not additive, and its strength is dependent on the point scatterer intensity [45]. For these reasons, the Poisson distribution is used to represent the likelihood function between the observation data and the observed scene in angular super-resolution, *i.e.*,

(18) $$p\left[g(k)|\left(Hf\right)(k)\right]=\frac{{\left[\left(Hf\right)(k)\right]}^{g(k)}\exp\left[-\left(Hf\right)(k)\right]}{\left(g(k)\right)!}$$

where (·) (*k*) represents the *k* -th element of the vector given by the expression inside the brackets.

Assuming that the values of **g**(*k*) are independent and identically distributed, the likelihood function of the data g is also Poisson, *i.e.*,

(19) $$p\left(g|Hf\right)\prod_{k=1}^{ℜ𝔄}\frac{{\left[\left(Hf\right)\left(k\right)\right]}^{g(k)}\exp\left[-\left(Hf\right)\left(k\right)\right]}{\left(g\left(k\right)\right)!}$$

### 3.2. Prior Law of the Targets

Since the echo formulation includes a convolution operation, some prior information about the statistical characteristics of the true scene must be introduced to regularize the solution of the deconvolution problem. In Bayesian theory, the prior information about the true scene is modeled as random variables with an assigned probability distribution, which represents a function of the the original scene and does not vary with the measurement.

The scanning radar image demonstrates the distribution and amplitude information of the point scatterers. Therefore, the dominant point scatterers can capture most of the information about the scene, and the weak scattering centers can be regarded as noise in the radar image. Note that the Laplace distribution has heavy tails, which means that the probability of strong scatters is large. Therefore, the statistical characteristics of strong scatters in the radar image can be modeled by an independent identical Laplace distribution with the same deviation [28,29,46,47]. As mentioned above, we utilize the Laplace distribution to represent the statistic characteristic of the dominant scatters in the scene. The law for f is also Laplace, so that the joint probability of f can be written as:

(20) $$p(f)=\prod_{i=1}^{ℜ𝔄}\frac{1}{\sqrt{2}\sigma }\exp\left(-\frac{\sqrt{2}\left|f(i)\right|}{\sigma }\right)$$

where σ denotes the deviation.

Substituting Equations (19) and (20) into Equation (17), the final expression for the MAP estimation task becomes:

(21) $$f_{\text{MAP}}=\underset{\text{f}}{\arg\max}\left\{\prod_{k=1}^{ℜ𝔄}\frac{{\left[\left(Hf\right)\left(k\right)\right]}^{g\left(k\right)}}{\left(g\left(k\right)\right)!}\exp\left[-\left(Hf\right)\left(k\right)\right]\right\}\times \left\{\prod_{i=1}^{ℜ𝔄}\frac{1}{\sqrt{2}\sigma }\exp\left(-\frac{\sqrt{2}\left|f(i)\right|}{\sigma }\right)\right\}$$

Maximizing Equation (21) is equivalent to minimizing:

(22) $$\begin{array}{ll} E(f) & =-\ln p\left(g|Hf\right)-\ln p(f)\\ & =\sum_{k=1}^{ℜ𝔄}\left\{\left(Hf\right)\left(k\right)-g\left(k\right)in\left[\left(Hf\right)\left(k\right)\right]\right\}+\frac{\sqrt{2}}{\sigma }{\left\|f\right\|}_{1} \end{array}$$

with respect to **f**. The term ${\left\|f\right\|}_{1}=\sum_{i=1}^{ℜ𝔄}\left|f(i)\right|$ is the *l*_1_-norm of *f* Note that we drop the ln [(**g**(k))!] term in the summation for the simplicity of notation, since this term has no effect on the corresponding minimization problem. From the point of the convex optimization, problem Equation (22) is equivalent to the MAP task Equation (21); solving Equation (22) for *f* yields the desired solution in Equation (17).

### 3.3. Solution of the Unconstrained Problem

The non-differentiability of the ‖ **f**‖ _1_ around the origin makes it impossible to solve Equation (22) by means of gradient-based optimization techniques. Fortunately, this problem can be solved by introducing a small positive parameter *ε* in the *l*_1_-norm (see [43,48]). Specifically, $\left|f\left(i\right)\right|\approx {\left[{\left|f\left(i\right)\right|}^{2}+ɛ\right]}^{\frac{1}{2}}$, where *ε* > 0 is a small constant. The role of *ε* is to ensure that the approximation is as rigid as possible; therefore, the parameter e should be set small. In this paper, we set the approximate parameter e to be 10^−8^. This modified l_1_-norm is a differentiable function, which enables us to solve the unconstrained problem Equation (22) within the framework of convex optimization by using the gradient-based approach. The first step in solving the unconstrained problem Equation (22) consists of replacing the *l*_1_*-norm* by its differentiable approximation. After the smoothed approximation, the ‖ **f**‖ _1_ has the following form:

(23) $$\left\|f_{1}\right\|\approx \sum_{i=1}^{ℜ𝔄}\left[{\left({\left|f\left(i\right)\right|}^{2}+ɛ\right)}^{\frac{1}{2}}\right]$$

Substituting Equation (23) into Equation (22), we express Equation (22) as follows:

(24) $$E\left(f\right)\approx \sum_{k=1}^{ℜ𝔄}\left\{\left(Hf\right)\left(k\right)-g\left(k\right)\ln\left[\left(Hf\right)\left(k\right)\right]\right\}+\frac{\sqrt{2}}{\sigma }{\sum_{i=1}^{ℜ𝔄}\left({\left|f\left(i\right)\right|}^{2}+ɛ\right)}^{\frac{1}{2}}$$

Since the objective function in the right-hand of Equation (24) is convex with respect to f, searching for a minimum is equivalent to searching for a zero of the gradient of Equation (24). This leads to:

(25) $$H^{T}I_{ℜ𝔄}-H^{T}\left(\frac{g}{Hf}\right)+\frac{\sqrt{2}}{\sigma }\Lambda \left(f\right)f=0$$

where the superscript *T* represents the transpose of a matrix, *I_ℜ 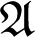
_* stands for *ℜ* 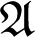 × 1 vector of ones and $\Lambda \left(f\right)=\text{diag}\left\{{\left[{\left|f\left(i\right)\right|}^{2}+ɛ\right]}^{-\frac{1}{2}}\right\}$ is a diagonal matrix whose *i*-th diagonal element is given by the expression inside the brackets. We further assume that **H***^T^I_ℜ 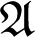
_* = *I_ℜ 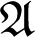
_*, where *I_ℜ 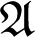
_* stands for the column vector consisting of *ℜ* 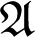 ones. *f_MAP_* solves the problem Equation (25) if and only if the following equivalent statements hold:

(26) $$H^{T}\left(\frac{g}{Hf_{\text{MAP}}}\right)-\lambda \Lambda \left(f_{\text{MAP}}\right)f_{\text{MAP}}=I_{ℜ𝔄}$$

where $\lambda =\frac{\sqrt{2}}{\sigma }$ is known as the regularization parameter, which controls the weight of the data term and the prior information of the observed scene. The method for selecting this parameter in Equation (26) is presented in the next section.

Solving Equation (26) above naturally calls for the fixed point iterative scheme; we can derive the following iteration form,

(27) $$f_{m+1}=f_{m}\left[H^{T}\left(\frac{g}{Hf_{m}}\right)-\lambda \Lambda \left(f_{m}\right)f_{m}\right]$$

where **f***_m_* and **f***_m_*_+1_ represent the estimationsof the true refllectivity coefficients of the observed scene **f** in the *m*-th and (*m* + 1)-th iterations, respectively. Assuming that at convergence, the ratio of **f***_m_*_+1_/**f***_m_* is *I*_*ℜ*
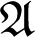
_. The greatest problem with multiplicative form Equation (27) is that the diagonal element ts of Λ (**f***_m_*) correspond to the approximation processes in the iteration.

## 4. Simulation and Experimental Results

In this section, we present experimental results to illustrate the angular super-resolution performance of the proposed deconvolution algorithm in forward-looking scanning radar. We compare the proposed deconvolution method with Wiener filter method, the Richardson-Lucy (R-L) algorithm and the Tikhonov regularization method.

### 4.1. Simulation

**Figure 3.**
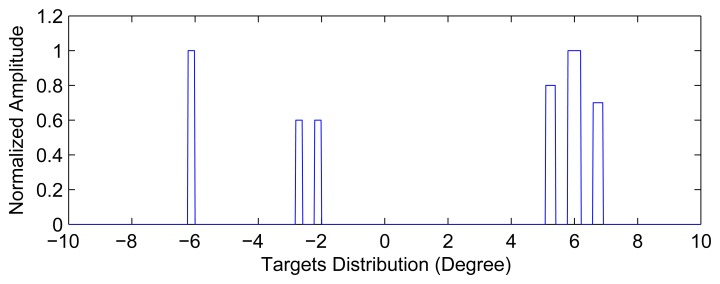
Location of targets in the simulated scene

For experiments on synthetic data, we apply our method to a synthetic scene composed of six point targets with different reflectivity magnitudes. The synthetic scene is shown in Figure 3. The targets are of unequal amplitude, which means different scattering coefficients. Some related radar parameters are set as follows: the pulse repetition frequency is 4000 Hz, and the antenna scanning speed is 30°/s. The bandwidth of the transmitted signal is 2 MHz, and the 3-dB width of the real beam is about 3°. The number of azimuth samples is 2666.

To provide a quantitative evaluation for the following super-resolution simulations, relative error (ReErr), improved signal-to-noise ratio (ISNR), structure similarity (SSIM) [49], and the signal-to-noise ratio (SNR) are used to measure the quality of the angular super-resolution results. They are defined as follows:

$$\begin{array}{ll} \text{SNR} & =20\log_{10}\frac{{\left\|f\right\|}_{2}}{{\left\|f_{\text{MAP}}-f\right\|}_{2}},\text{ReErr}=\frac{{\left\|f_{\text{MAP}}-f\right\|}_{2}}{{\left\|f\right\|}_{2}}\\ \text{ISNR} & =20\log_{10}\frac{{\left\|g-f\right\|}_{2}}{{\left\|f_{\text{MAP}}-f\right\|}_{2}}\\ \text{SSIM} & =\frac{2\rho_{\left(f_{\text{MAP}},f\right)}\cdot \left(2\mu_{f_{\text{MAP}}}\cdot \mu_{f}\right)}{\left(\mu_{f_{\text{MAP}}}^{2}+\mu_{f}^{2}\right)\left(\sigma_{f_{\text{MAP}}}^{2}+\sigma_{f}^{2}\right)} \end{array}$$

where **f***_MAP_*, **f** and **g** correspond to the obtained angular super-resolution image, the original image and the observed image, respectively The terms μ, σ, and ρ_(_**_f_** ,**_f_**_*MAP*)_ are the mean and standard deviation of the vectors, and the correlation coefficient corresponds to the vector **f** and **f***_MAP_*. The SSIM is a quantitative measure between the super-resolution result and the original scene. The value of SSIM is between −1 and one, and one means fully identical to the original scene.

In order to show the performance of the proposed deconvolution algorithm for angular super-resolution under different noise levels, Gaussian noise models the signal-independent part of the errors, such as thermal and electric noise, which is stronger than the signal-dependent noise in scanning radar imaging. Using the approximation *N*(σ ^2^, σ ^2^) = *Poiss*(σ^2^), the Poisson noise can be approximated by Gaussian noise. Then, the Gaussian noise with different levels is added to model the errors.

#### 4.1.1. Parameter Values

In most of the Bayesian deconvolution algorithms, the regularization parameter has to be chosen, so that it gives the best visual results. Both the data fidelity and the prior are presented in Equation (22), and their size depends on the regularization parameter λ. Small values of the regularization parameter tend to amplify the noise, while a large regularization parameter over smooths the radar image. Most of the referenced methods need to manually choose the regularizing parameter λ to control the weight of the prior, so that the result of super-resolution gives the best visual quality. However, this approach is time consuming and relies too much on subjective factors. To overcome this problem, a number of works on the selection of regularization parameter, such as the *L*-curve method [50], generalized cross-validation [51] and the unbiased predictive risk estimator method [52], have been reported.

In order to determine the parameter λ in Equation (27), we choose the regularization parameter λ by means of the *L*-curve method, which plots the squared norm of the prior solution *X*(λ) against the squared norm of the corresponding residual solution *Y*(λ). The corner of the regularization parameter curve is identified, and the corresponding parameter value λ is picked out as the weight coefficient in the deconvolution process Equation (27). Specifically, *L*-curve method is proposed in [50], which selects the regularization parameter A that maximizes the curvature function:

(28) $$C\left(\lambda \right)=\frac{X^{\prime \prime }\left(\lambda \right)Y^{\prime }\left(\lambda \right)-X^{\prime }\left(\lambda \right)Y^{\prime \prime }\left(\lambda \right)}{{\left[X^{\prime }{\left(\lambda \right)}^{2}+Y^{\prime }{\left(\lambda \right)}^{2}\right]}^{\frac{3}{2}}}$$

where X(λ) = log(‖Hfλ−g‖2) and *Y*(λ) = log(‖*D*(|fλ| ‖2) are the log-transform of the data fidelity and the prior information, respectively, and the superscript (′)erepresents the differentiation with respect to λ

**Figure 4.**
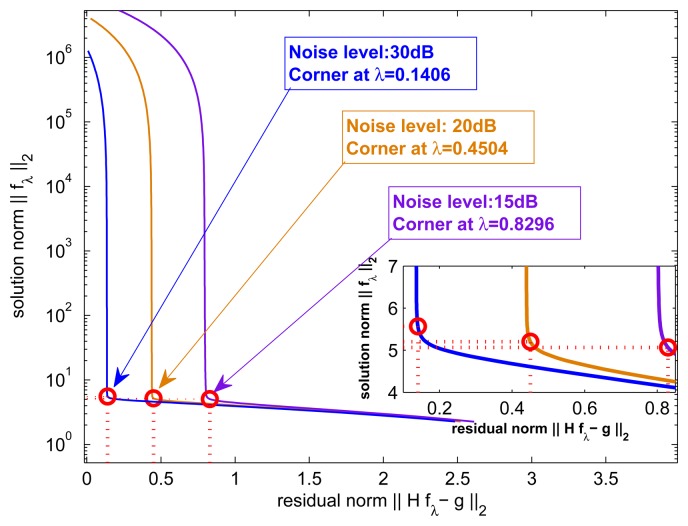
The *L*-curve at the different noise levels; the corner corresponds to the point with the maximum curvature.

In the simulations, the parameter λ is selected using the *L*-curve method. This is owed to the advantage the *L*-curve method is able to treat perturbations consisting of corrected noise and robustness no matter which deconvolution method is used. The *L*-curve method is useful for direct solvers. However, there are two parameters in the solution obtained using the proposed deconvolution algorithm. This paper focuses on the performance of the proposed deconvolution algorithm in terms of angular super-resolution in scanning radar. The automatic choice of the regularization parameter λ beyond the scope of this paper. To determine a good pair of parameters in Equation (27), we firstly use the iterative solver with a lot of iterations and re-use it for all of the different choices of the regularization parameter λ Then, we choose the good solution using the *L*-curve. Figure 4 shows the obtained λ under different noise levels, which is used in the following simulations. The setting of the iteration number of the R-L algorithm is equal to the way for the proposed algorithm.

#### 4.1.2. Angular Super-Resolution Results

Figures 6, 7 and 8 show the angular super-resolution results under different noise levels. The $\text{BSNR}=20\log_{10}\frac{{\left\|g\right\|}_{2}}{{\left\|n\right\|}_{2}}$ is used to measure the quality of these echo data with different noise levels. The regularization parameter λ is computed by using Equations (28), and Figure 4 shows the *L*-curve and the corresponding curvature as a function of λ. The number of iterations in Figure 4 are 75,110 and 150, respectively.

**Figure 5.**
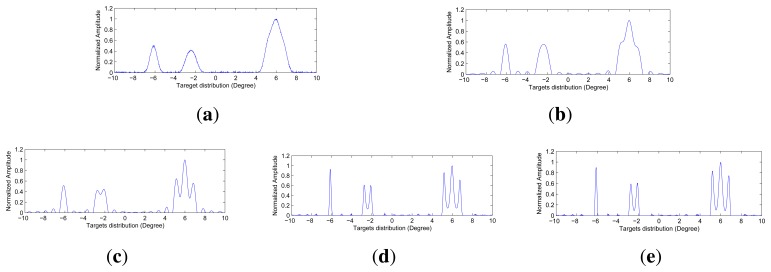
(**a**)The echo data added by Gaussian noise with 30 dB; (**b**) Angular super-resolution result of the Tikhonov regularization; (**c**) Angular super-resolution result of the Wiener filter; (**d**) Angular super-resolution result of the R-L algorithm with 75 iters; (e) Angular super-resolution result of the proposed method with 75 iters and λ =0.1406.

**Figure 6.**
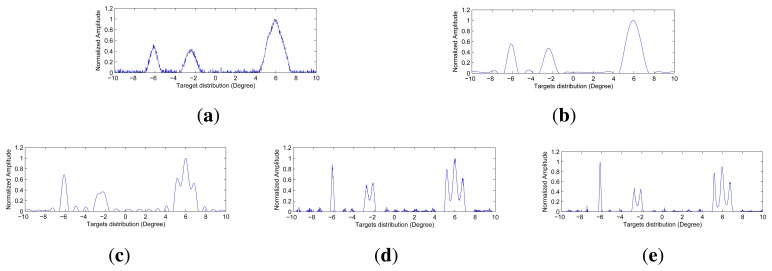
(**a**) The echo added by Gaussian noise with 20 dB; (**b**) Angular super-resolution result of the Tikhonov regularization; (**c**) Angular super-resolution result of the Wiener filter; (**d**) Angular super-resolution result of the Richardson–Lucy (R-L) algorithm with 110 iters; (e) Angular super-resolution result of the proposed method with 110 iters and λ =0.4504.

**Figure 7.**
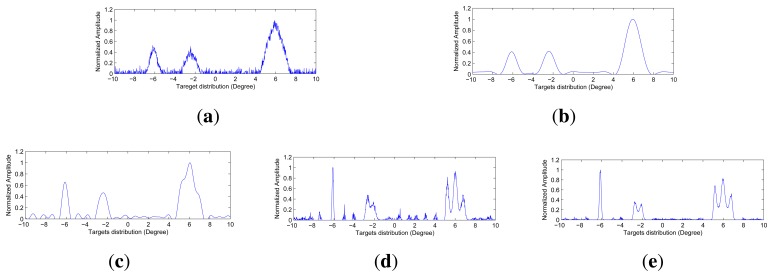
**(a)** The echo added by Gaussian noise with 15 dB; (b) Angular super-resolution result of the Tikhonov regularization; (c) Angular super-resolution result of the Wiener filter; (d) Angular super-resolution result of the R-L algorithm with 150 iters; (e) Angular super-resolution result of the proposed method with 150 iters and λ = 0.8296.

The angular super-resolution results by those methods under various noise levels are shown in Figures 5, 6 and 7. The visual quality of super-resolution results using the proposed deconvolution algorithm are quite competitive with those using the Tikhonov regularization method, Wiener filter method and R-L algorithm. It is noted that the angular super-resolution results by using the proposed deconvolution algorithm exhibit a close match with the original scene.

It can be seen from Figures 5, 6 and 7 that the spikes of the targets in the results of the Tikhonov regularization and Wiener filter are more connected compared with the results of the R-L algorithm and the proposed method. The reason for this is that the Tikhonov regularization method is based on the noise power of the observed scene, which makes the profile of targets overly smooth. The Wiener filter obtains an optimal result in the sense of minimizing the mean square error between the obtained result and the true scene using the correlation information between the signal and noise. Therefore, the angular super-resolution performance of the Wiener filter is degraded when the received signal includes complicated targets and high noise.

In Figure 5, we can see that the visual quality of the angular super-resolution result by using the R-L algorithm looks similar to the result by using the proposed method. In Figure 6, the proposed approach gives the super-resolution result, where the spikes of the targets look fairly separate, whereas in the angular super-resolution result, by using the R-L algorithm, the spikes of the targets looks more connected. The improvement of the proposed method compared to R-L algorithm can also be appreciated in Figure 7, as the spikes of the targets have been separated, and the noise amplified by the R-L algorithm is not presented in the result by using the proposed method.

The reason is that the R-L algorithm is based on the maximum likelihood criterion, which is identical to the deterministic method with no penalty function or prior information. The maximum likelihood criterion aims at maximizing the agreement between the measurement and the object, which yields high noise estimates, particularly when the noise level is low. This conclusion is also supported by Table 1, in which the evaluation parameters of the proposed algorithm are better than the evaluation parameters associated with the comparative methods.

Table 1 shows the comparisons between the Tikhonov regularization method, Wiener filter, R-L algorithm and the proposed deconvolution method on super-resolution results in SNRs, ISNRs and SSIMs. Referring to Table 1, it is shown that the proposed deconvolution algorithm produces the highest SSIM values while keeping ISNR at a high level. Among the four methods, both the Tikhonov regularization method and the Wiener filter use the inverse filtering, which gives poor results. The R-L algorithm may be an attractive deconvolution approach for super-resolution imaging, but the noise is amplified after a small number of iterations, and the false targets emerge. This phenomenon is a generic problem for the R-L algorithm, leading to the larger error of the corresponding angular super-resolution result. These results demonstrate that the proposed deconvolution algorithm for angular super-resolution is quite competitive over the conventional deconvolution algorithms.

**Figure 8.**
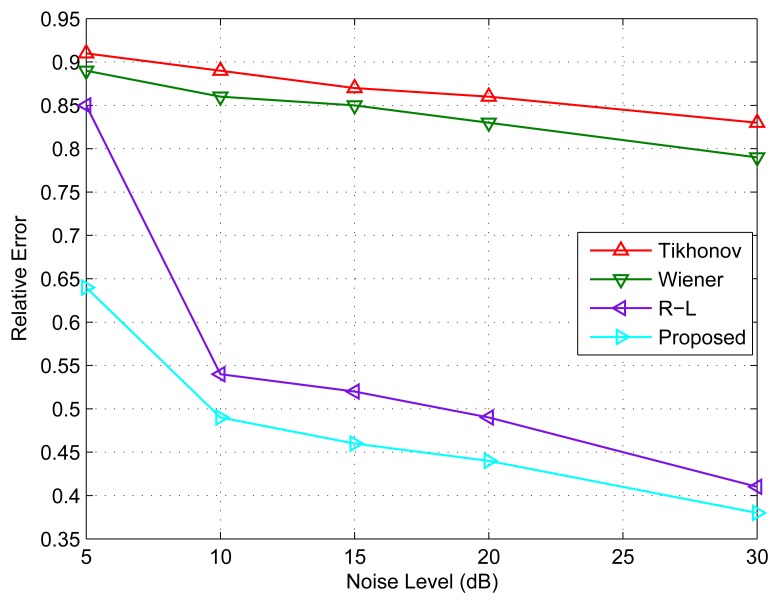
Relative error performance comparison under various noise levels.

In addition, another noticeable advantage of the proposed deconvolution algorithm compared to conventional deconvolution algorithms is that the proposed deconvolution algorithm suppresses the spurious peak appearance in the angular-resolution results under different noise levels, which leads to a better angular super-resolution performance in terms of precision. Figure 8 shows the relative error performance of the algorithms in the different noise levels. It can be seen from Figure 8 that the Tikhonov regularization method and Wiener filter have higher relative errors under various SNR levels, while the proposed deconvolution algorithm is much better than the conventional algorithms in terms of precision. The reason is that the proposed deconvolution algorithm has explored the prior information about the targets. This leads to a more stable solution to the associated deconvolution problem, and the evaluation parameters are also better than other comparative methods.

### 4.2. Experiment

**Figure 9.**
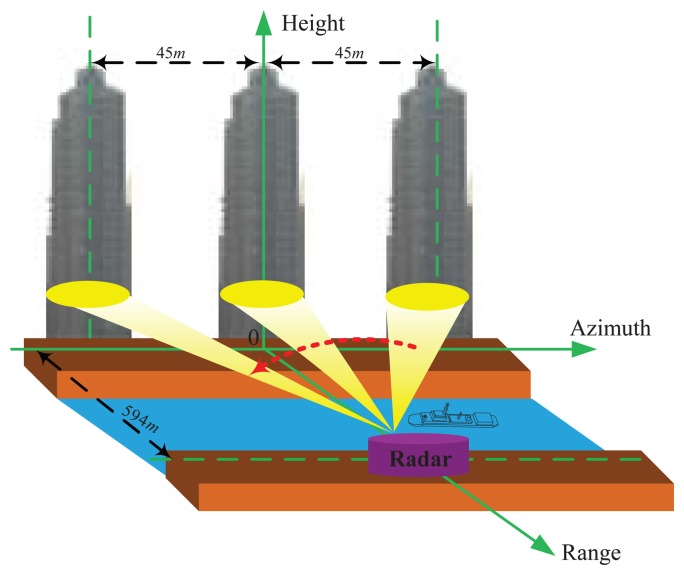
The tested scene.

**Figure 10.**
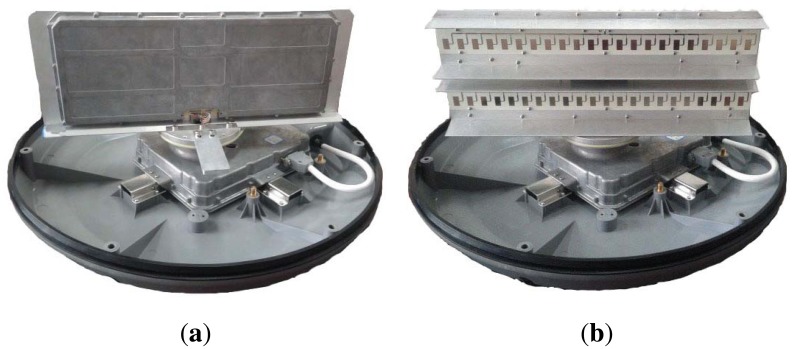
The scanning radar system ((**a**) front side and (**b**) back side) utilized to collect scattering data.

In order to show the validity of the proposed method, we present the real data results. Figure 9 shows the tested scene in which three buildings and their distribution are shown. Employing the scanning radar system in Figure 10, the real data are acquired. The scanning radar system parameters for the experimental result are shown in Table 2. The resolution of the range dimension was calculated to be 1 m. The distance of each building is 45 m. The range from the scene center to the radar system is about 594 m, and the angular resolution in the scene center is 95 m. The echo from three buildings is much stronger than that from the other areas of the observed scene, so that the scene can be consider as consisting of three main scattering point targets.

**Figure 11.**
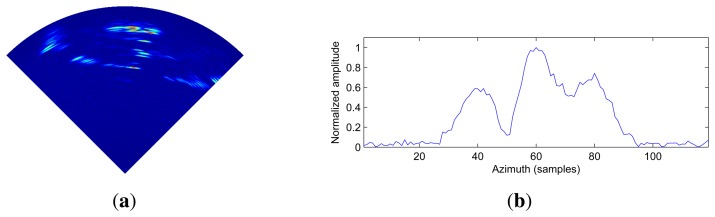
**(**a**)** The received echo signal of the tested scene after range compression; (b) The profile of the target ares.

The range compression is applied to the recorded data. The imaging result and the corresponding profile of the target area are shown in Figure 11a,b, respectively. From Figure 11b, we can see that the echo of adjacent buildings is overlapping and covering the building features.

As compared with other methods, the maximum iterations and the regularization parameter of the proposed algorithm are hand-tuned alternatives, so that the corresponding super-resolution result presents the best visual quality. For the proposed deconvolution algorithm, we used the regularization parameter λ = 1.47, and the iteration number is 100.

**Figure 12.**
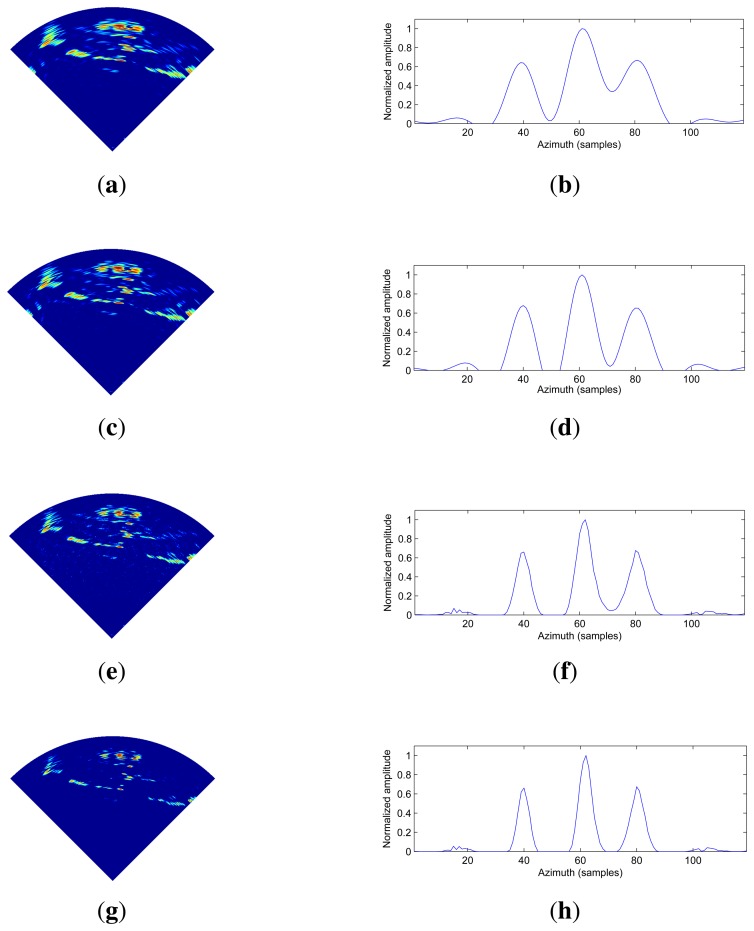
The results of the angular super-resolution using different methods, (**a**) Angular super-resolution result obtained using Tikhonov regularization method; (**b**) The profile of Angular super-resolution result obtained using Tikhonov regularization method in the target area; (**c**) Angular super-resolution result obtained using Wiener filter method; (**d**) The profile of Angular super-resolution result obtained using Wiener filter method in the target area; (**e**) Angular super-resolution result obtained using R-L algorithm; (**f**) The profile of Angular super-resolution result obtained by using Wiener filter method in the target area; (**g**) Angular super-resolution result using the proposed method; (**h**) The profile of Angular super-resolution result obtained using proposed method in the target area.

In Figure 12, we investigate the angular super-resolution performance of the approaches. The first column of Figure 12 gives the angular super-resolution imaging results of different methods. The right column of Figure 12 presents the profile of the target scene corresponding to the left column. The first row presents the angular super-resolution results using the Tikhonov regularization method. The second row contains the Wiener filter results, while the third and bottom rows contain angular super-resolution results by using the R-L algorithm and the proposed algorithm, respectively. It can be obviously observed that the amplitudes of the target profiles in the right column of Figure 12 are different. The reason for this is that the distance from the middle building to the radar is about 594 m, while the distance between the radar and the left/right building is about 597 m. This leads to the profile of the middle building being higher than the other two profiles in the right column of Figure 12, which also fits their physical truth.

The left column of Figure 12 shows the angular super-resolution imaging results. It can be noted that the proposed method gives the best results in terms of visible quality. This is due to the fact that the proposed method is able to suppress the noise amplification by incorporating the prior information about the targets. We can see that the angular super-resolution results obtained by using the Tikhonov regularization method and Wiener filter method are still noisy, particularly around the building boundaries. The improvement of the proposed method compared to the other methods can also be appreciated in the right column of Figure 12. The spikes of buildings in the result of the proposed method look fairly separated, whereas the spikes of buildings in the other three results look more connected. Therefore, we believe that the proposed method for angular super-resolution in scanning radar is useful in real applications.

## 5. Conclusions

The angular super-resolution in scanning radar has received much attention in recent years; however, limited work about the application of the deconvolution algorithm for angular super-resolution in scanning radar has been reported. Since the conventional linear deconvolution approaches result in large noise amplification and, thus, a poor angular resolution, this paper proposes a deconvolution algorithm for angular super-resolution in scanning radar. This algorithm can be interpreted as solving a deconvolution problem, corresponding to the original angular super-resolution problem under the MAP criterion. To use the MAP criterion for realizing the angular super-resolution, we first transform the original angular super-resolution problem into an equivalent maximum a *posteriori* estimation task, so that the prior information about the statistical characteristics of the original scene can be incorporated. In this paper, the Laplace distribution is used to represent the prior information about the targets. This makes the resulting MAP estimation task challenging due to the presence of a “non-smooth” function in the cost function, which calls for an effective and robust differentiable approximation. Therefore, a convex optimization method with differentiable approximation is employed to solve the associated MAP estimation task. In our experiments with synthetic data, the proposed deconvolution algorithm for angular super-resolution has higher precision and suppresses the noise amplification in the angular super-resolution results.

## Figures and Tables

**Table 1.** Summary of angular super-resolution results for different methods. ISNR, improved signal-to-noise ratio; SSIM, structure similarity.

**Image**	**Method**	**SNR (dB)**	**ISNR (dB)**	**SSIM**
	Tikhonov	2.93	−0.43	0.61
Figure 5a	Wiener	3.93	1.43	0.71
BSNR = 14.91 dB	R-L	7.71	5.20	0.89
	Proposed	8.45	5.94	0.91

	Tikhonov	2.45	0.02	0.54
Figure 6a	Wiener	3.17	0.69	0.64
BSNR = 9.94 dB	R-L	6.28	3.74	0.83
	Proposed	7.15	4.66	0.88

	Tikhonov	2.30	0.05	0.50
Figure 7a	Wiener	2.83	0.47	0.58
BSNR = 7.69 dB	R-L	5.60	3.19	0.80
	Proposed	6.71	4.36	0.84

**Table 2.** Experimental parameters for real data.

**Parameters**	**Value**	**Units**
Carrier frequency	10	GHz
Band width	75	MHz
Pulse duration	2	U-S
Sampling frequency	200	MHz
Antenna scanning velocity	70	°/s
Antenna scanning area	−45 ± 45	°
Main-lobe beam width	5	°
